# An Aberrant Microbiota is not Strongly Associated with Incidental Colonic Diverticulosis

**DOI:** 10.1038/s41598-018-23023-z

**Published:** 2018-03-21

**Authors:** Roshonda B. Jones, Anthony A. Fodor, Anne F. Peery, Matthew C. B. Tsilimigras, Kathryn Winglee, Amber McCoy, Michael Sioda, Robert S. Sandler, Temitope O. Keku

**Affiliations:** 1 Department of Bioinformatics and Genomics, University of North Carolina, Charlotte, NC USA; 2 Center for Gastrointestinal Biology and Disease, University of North Carolina, Chapel Hill, NC USA

## Abstract

Colonic diverticula are protrusions of the mucosa through weak areas of the colonic musculature. The etiology of diverticulosis is poorly understood, but could be related to gut bacteria. Using mucosal biopsies from the sigmoid colon of 226 subjects with and 309 subjects without diverticula during first-time screening colonoscopy, we assessed whether individuals with incidental colonic diverticulosis have alternations in the adherent bacterial communities in the sigmoid colon. We found little evidence of substantial associations between the microbial community and diverticulosis among cases and controls. Comparisons of bacterial abundances across all taxonomic levels showed differences for phylum Proteobacteria (p = 0.038) and family Comamonadaceae (p = 0.035). The r-squared values measuring the strength of these associations were very weak, however, with values ~2%. There was a similarly small association between the abundance of each taxa and total diverticula counts. Cases with proximal only diverticula and distal only diverticula likewise showed little difference in overall microbiota profiles. This large study suggests little association between diverticula and the mucosal microbiota overall, or by diverticula number and location. We conclude that the mucosal adherent microbiota community composition is unlikely to play a substantial role in development of diverticulosis.

## Introduction

Colonic diverticulosis and diverticular-related disorders are common in the United States. More than 50% of individuals over the age of 60 are estimated to have diverticulosis^1^. Colonic diverticula form when mucosa and submucosa herniate through the muscularis propria^2^. Colonic diverticula can be complicated by acute inflammation, infection, hemorrhage and there is some evidence for a spectrum of chronic diverticula-related bowel disorders. The economic burden of acute diverticular-related disorders is estimated at $4 billion dollars annually in the United States^3^. The burden is likely to increase as the population ages^2,4^.

The etiology of diverticulosis is not known. For many years, a high fiber diet was thought protective, largely based on ecologic studies by Painter and Burkett^5,6^. However, contemporary colonoscopy-based studies have cast doubt on the fiber hypothesis^7,8^. The gut microbiota could plausibly be related to diverticulosis. The bacterial flora is important for the function and integrity of the intestinal epithelial barrier and its blood supply, and is essential for the development of gut motility^9^. Local quantitative and qualitative alterations in gut microbes could potentially induce inflammatory or neuromuscular changes associated with diverticulosis. There is very limited information about the association between diverticulosis and bacterial communities in the large bowel. In a pilot study with 16 diverticulosis cases and 14 controls Barbara *et al*.^10^ compared the fecal and mucosal microbiota and found no significant differences in the mucosal microbiota between controls and patients with asymptomatic diverticulosis in the sigmoid and proximal colon. There were differences seen in the 8 patients with symptomatic uncomplicated diverticular disease (SUDD).

An imaging study is necessary to correctly classify an individual as having colonic diverticulosis. With the widespread use of screening endoscopy for colorectal cancer in the United States, we now have the opportunity to identify large numbers of patients with colonic diverticulosis and obtain specimens for microbial analysis. To assess whether individuals with incidental colonic diverticulosis have alterations in microbial communities, we examined the adherent mucosal bacterial communities in the sigmoid colon in a large group of patients with and without diverticulosis.

## Results

We evaluated the role of the microbiota in colonic diverticulosis among 226 patients with diverticulosis and 309 diverticulosis-free controls. As previously reported^11^, participants with diverticula were more likely to be older, male, and have a higher body mass index than those without diverticula (Table 1).

**Table 1:** Participant characteristics.

	Diverticula	No diverticula
**n (%) or mean ± standard deviation**	n = 226	n = 309
**Age**		
≤49 y	19 (8)	32 (10)
50–59	150 (66)	231 (75)
60–69	48 (21)	41 (13)
70–79	8 (3)	8 (2)
≥80	1 (0.4)	0 (0)
**Sex**		
Male	106 (47)	122 (39)
Female	120 (53)	187 (61)
**Race**		
White	172 (76)	233 (75)
Black	44 (19)	62 (20)
Other	1 (0.4)	8 (3)
**Smoking status**		
Never	91 (40)	157 (60)
Former	55 (24)	60 (19)
Current	22 (10)	26 (8)
**Body mass index, kg/m** ^**2**^		
Underweight (<18.5)	5 (2)	6 (2)
Normal (18.5–25)	58 (26)	105 (34)
Overweight (25–30)	73 (32)	93 (30)
Obese (>30)	90 (40)	101 (33)
Waist circumference, centimeters	97.4 ± 17.4	93.2 ± 15.8
**Number of diverticula**		
1–3 diverticula	59 (26)	—
4–10 diverticula	85 (38)	—
≥10 diverticula	82 (36)	—
Location of diverticula		
Proximal only	14 (6)	—
Distal only	139 (63)	—
Proximal and distal	69 (31)	—

**Figure 1:**
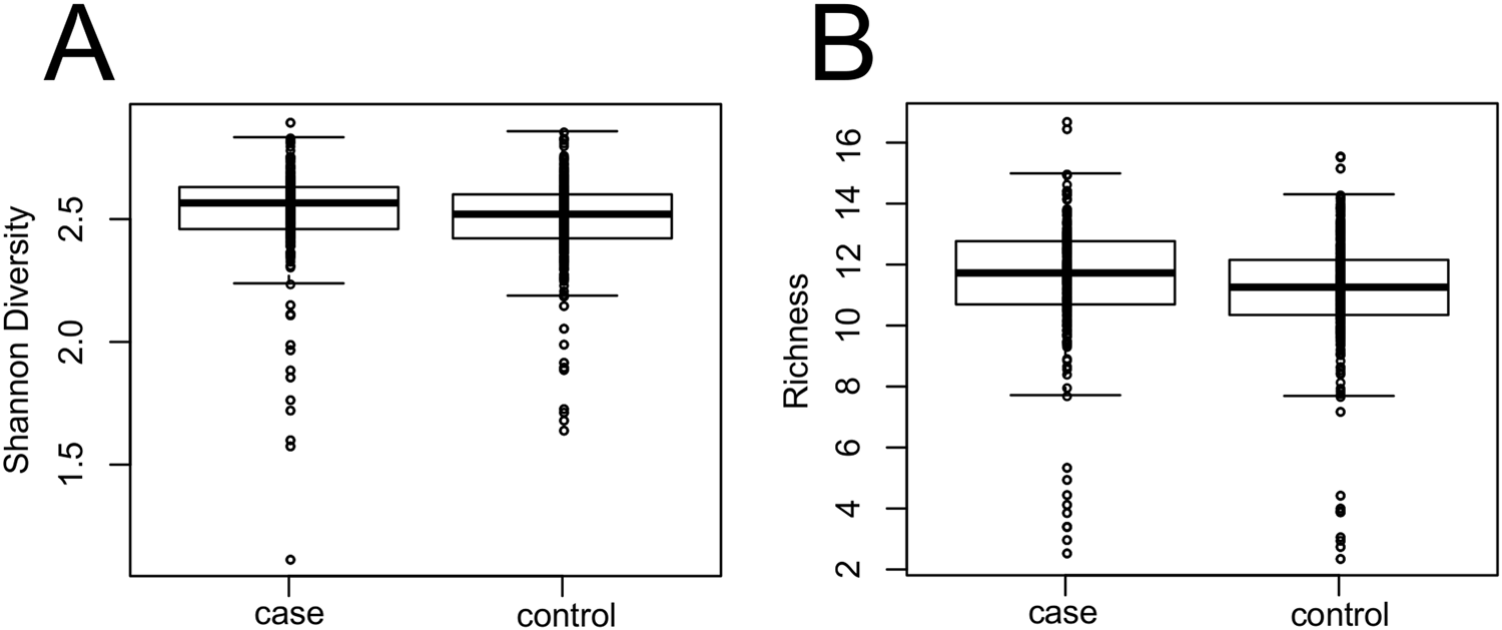
Shannon diversity and richness show minor differences. Shannon diversity (**A**) and richness (**B**) for the 226 case and 309 controls subjects in our study. FDR corrected p-values = 0.011 and 0.012 from Wilcoxon test respectively at the class taxonomic level with r-squared values (determined by linear model) <1% (Supplemental Table 1).

**Figure 2:**
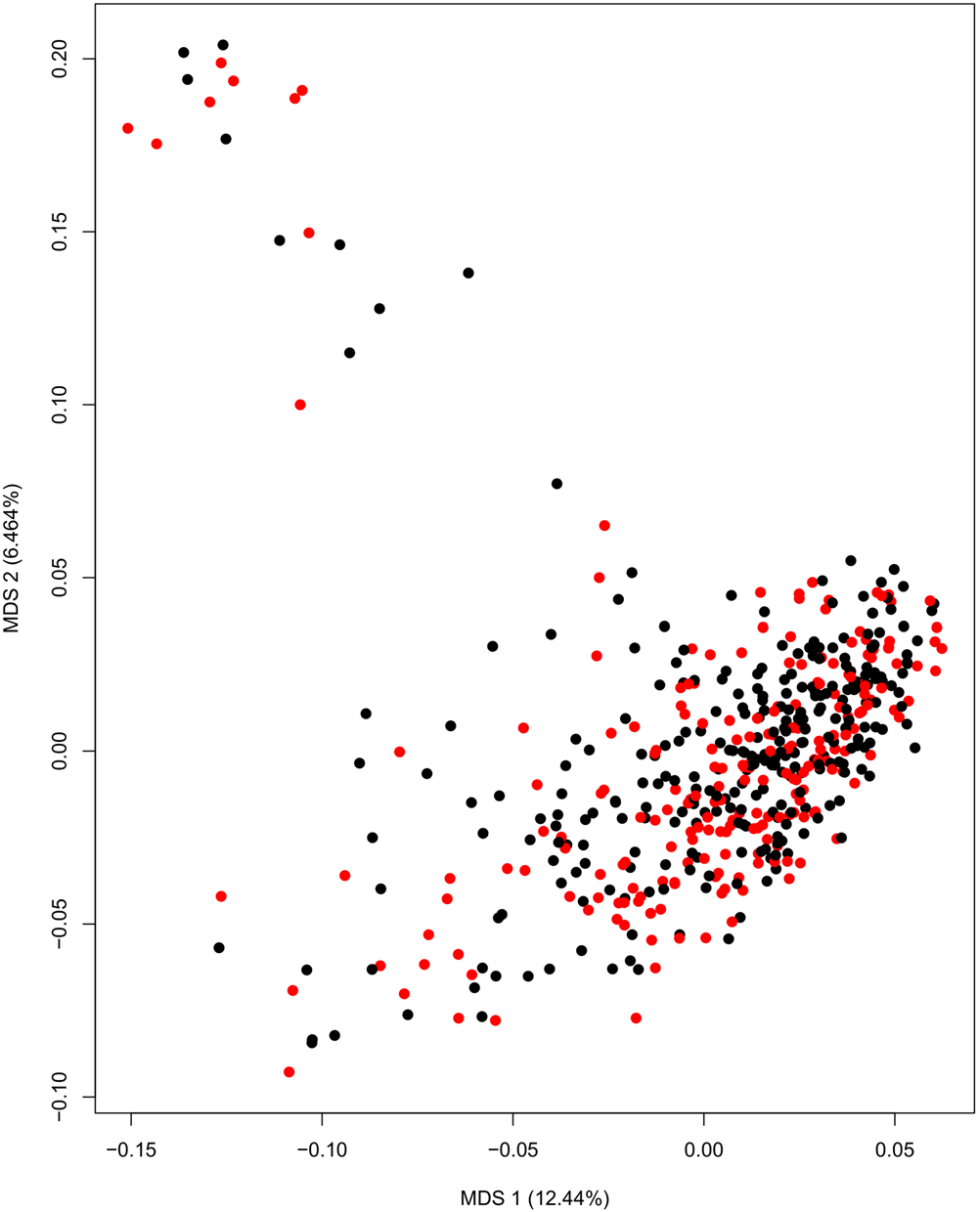
MDS ordination at the genus level shows little difference between cases (red) and controls (black). Ordination based on Bray-Curtis dissimilarity. Neither the first nor the second MDS axis differed significantly between cases and controls (p > 0.05, unpaired Wilcoxon test).

**Figure 3:**
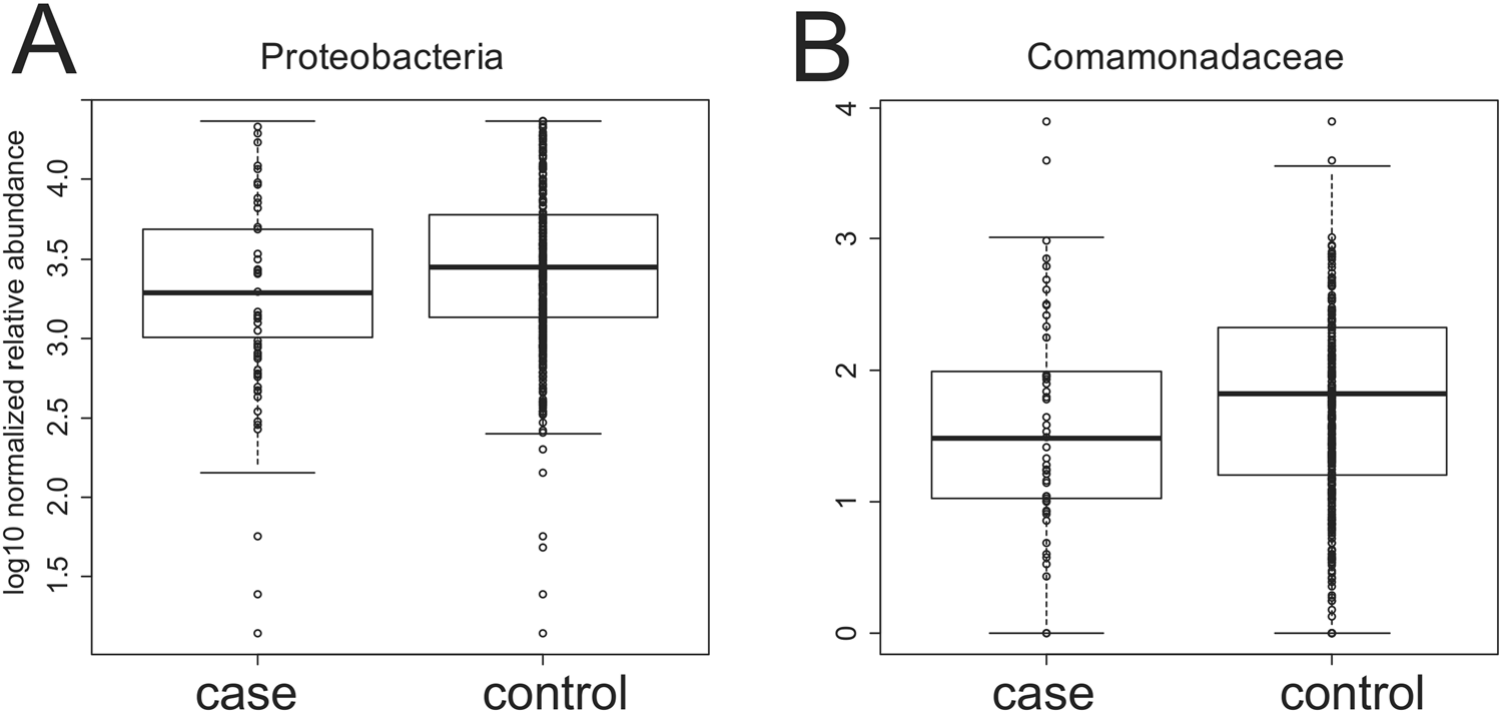
The microbial community shows only very modest associations with diverticulosis status. Comparison of the two taxa (Table 3; Supplementary Tables 2–6) that were significantly associated with case-control status. For both panels, FDR corrected p-values are p < 0.05; r-squared values (determined by linear model) were <0.02 (Table 3).

In general, we found very limited to no associations between the microbiota profiles and the presence of diverticulosis. Across taxonomic levels, Shannon diversity was only significantly associated with diverticulosis case-control status at the class level p = 0.012 (FDR corrected Wilcoxon), but with an associated effect size of <1% (r-squared from Pearson correlation). Similarly, the only association between richness and diverticulosis case/control was at the class level (p = 0.011; FDR corrected Wilcoxon) again with a r-squared <1%, (Supplementary Table 1, Fig. 1). Likewise, multidimensional scaling ordination (MDS) (Fig. 2) revealed no statistically significant differences between diverticulosis cases and diverticula-free controls. We also performed analysis at each phylogenetic level to test the null hypothesis of no association of each taxon with the presence of diverticulosis (Supplementary Tables 2–6). Across all taxonomic levels, phylum Proteobacteria and family Comamonadaceae were the only two taxa that had significant associations at a 5% FDR threshold (Table 2). Even for these taxa, the r-squared values measuring the strength of the association were very weak with values ~2% (Table 2, Fig. 3). We conclude that even though our large sample size allowed us to find some associations, the strength of these associations is very modest despite over 500 total patients in our cohort.

**Table 2:** All taxa significant at an FDR corrected value of p < 0.05 across all taxonomic levels comparing case and control status.

Names	p Values	r-Squared	Adjusted p-value	Tax. Level
Proteobacteria	0.004	0.022	0.038	Phyla
Comamonadaceae	0.0006	0.018	0.035	Family

*P-values (non-parametric Wilcoxon test). R-squared values are from linear models. N = 220, case; N = 295, control. See Supplementary Tables 1–5 for results of all statistical tests.

We were concerned that the lack of association might be because of the coarse assignment of case-control to patients who might have a range of disease severity. We therefore compared the abundance of each taxa to the total count of diverticula from each patient. At an FDR-adjusted threshold of p < 0.05, three taxa (Table 3) were significantly associated with the diverticula count, but again the effect size were very modest with r-squared values ~1%. We conclude that using the diverticula count rather than a binary case-control assignment did not substantially improve our power.

**Table 3:** All taxa significant at an FDR corrected value of p < 0.05 across all taxonomic levels comparing the log-normalized abundance of each taxa to diverticula count.

Names	pValues	rSquared	Adjusted p-value	Tax.level
Crenarchaeota	6.29E-03	8.92E-03	3.15E-02	phyla
Synergistetes	4.42E-03	1.09E-02	3.15E-02	phyla
Comamonadaceae	4.47E-04	1.31E-02	2.50E-02	family

*P-values (non-parametric Kendall test). R-squared values are from the Kendall correlation statistic. Data from subjects for which diverticula counts was available. See Supplementary Tables 1–5 for results of all statistical tests.

We next asked whether the location of the diverticula made a difference. We separately examined the subset of patients who had diverticula in only the distal or only the proximal colon. At a 5% FDR cutoff, there were only two taxa across all taxonomic levels (genus *Hallella* and *Delftia*) that showed significant differences in patients with distal or proximal diverticula (Supplemental Table 5; n = 135 distal, n = 14 proximal). For both of these taxa, the r-squared value of the association with location was <4%. We conclude that diverticula location did not have a strong effect on the microbial community, although we may have limited power to address this question due to the small number of patients with only proximal diverticula.

In addition to diverticulosis, we examined associations with a number of patient metadata (Supplemental Tables 2–5). Associations with sex and race were slightly stronger than the associations with diverticulosis. There were 25 significant taxa associated with sex (Supplemental Table 8) and 40 taxa associated with ethnicity (Supplemental Table 9) at a 5% FDR. While these hits are stronger associations than we saw with diverticulosis, they were quite modest with r-squared values of 2–3% and no taxa showing an r-squared of >6%. Correlations with waist circumference were much more modest with only two significant taxa (phylum Verrucomicrobia and genus *Asaccharobacter*) both of which had r-squared values of 5%. Only one taxa (class “Deltaproteobacteria”) was significantly associated with age (p < 0.05). We conclude that, as has been observed in other large cohorts^12,13^, associations of patient metadata with the composition of the microbiota are modest.

## Discussion

Colonic diverticulosis is common and the complications are costly. Because complications such as diverticulitis can only occur in patients with diverticulosis, if we could uncover the etiologic risk factors for diverticula, we could potentially prevent complications. In this large study, we found little to no difference in microbial composition between individuals with and without diverticula. Based on the large size of this study and the small effect sizes we observed, it is not likely that changes in bacterial relative abundance are responsible for the development of colonic diverticula. In addition, the presence of diverticulosis does not alter the microbial composition to a significant degree.

Although bacteria have been associated with a number of gastrointestinal disorders, prior information on a bacterial etiology for colonic diverticula is limited. A pilot study of 38 subjects from Italy examined bacteria profiles in feces and mucosal biopsies^10^. Compared to controls, the patients with diverticulosis had a lower relative abundance of Clostridium cluster IV bacteria, although the difference was not statistically significant. The general microbiota composition in colonic biopsies showed no significant differences between controls and diverticulosis patients. There was a lower abundance of Enterobacteriaceae in the diverticulosis cases compared to controls and a non-significant higher abundance of *Bacteroides/Prevotella*.

It should be stressed that this was a study assessing the microbiome of patients with incidental colonic diverticula. This is not a study of the microbiome in patients with complications of colonic diverticulosis. While a proportion of our population reported symptoms of irritable bowel syndrome and chronic abdominal pain, there is no evidence that these symptoms are associated with colonic diverticulosis, so called symptomatic uncomplicated diverticular disease (SUDD). Our group recently published a colonoscopy-based study that found no association between colonic diverticulosis and chronic gastrointestinal symptoms or mucosal inflammation^14^. As such, we did not assess the microbiome in patients with colonic diverticulosis and chronic symptoms.

While we found no differences in the gut microbiota between individuals with asymptomatic diverticulosis (AD) and healthy controls, diverticulosis represents a continuum in the progression to diverticular disease. Therefore, we cannot exclude the role of the gut microbiota in the disease progression. Several small studies have reported alterations in the gut microbiota in SUDD patients^15–17^. Tursi *et al*.^18^ evaluated the fecal microbiota in SUDD patients, diverticulosis patients and healthy controls. They found no overall differences in bacterial abundances between the three groups but the levels of fecal *Akkermansia muciniphila* was significantly higher in diverticulosis and SUDD patients. Another study found higher bacterial diversity and increased abundance of *Proteobacteria* in diverticulitis patients compared to controls^15^. One study assessed bacteria and fungi in diverticulitis tissue from the sigmoid colon and adjacent unaffected tissue. They observed an enrichment of *Microbacteriaceae and Ascomycota* in diverticulitis tissue^17^ suggesting that the diverticulum microbiota may be different from adjacent mucosa. These studies implicate the gut microbiota in diverticulitis, but larger studies are needed to confirm their findings. In our study, we assessed the gut microbiota (bacteria) but we did not evaluate the fungal mycobiome because it is an emerging field that was not well characterized until recently.

Our large sample size revealed some borderline significant associations, but there was little evidence of a strong association with diverticulosis. As with any negative results, we might have seen stronger association with different methods (RNA-seq, metabolomics, whole-genome metagenome shotgun sequencing). If we had corrected for multiple hypothesis testing including all hypotheses in one correction, nothing in our paper would have been significant. This again emphasizes the modest nature of the associations that we observed.

We chose to examine mucosal adherent bacteria from biopsies rather than feces. It was logistically simple and safe to obtain biopsies from patients during their colonoscopy. More importantly, although there are known differences in the bacterial composition of feces and mucosal biopsies^19^, we reasoned that the adherent bacteria would be more likely to influence the colonic mucosa. All patients in the study underwent a colonoscopy prep that could change the bacterial composition. Adherent bacteria are less influenced by a purge and all patients in the study were prepped^20^.

This paper has notable strengths. All subjects underwent their first colonoscopy for screening purposes rather than colonoscopy for symptoms that might be associated with diverticulosis. We systematically recorded diverticula from all colon segments. Mucosal associated bacteria were evaluated from biopsies from the sigmoid colon. The biopsies were handled in a uniform manner by technicians who were blinded to diverticulosis status. Importantly, the sample size was very large.

Because the patients were drawn from a single academic medical center in the US, the results may not be widely generalizable. The pilot study by Barbara *et al*. reported differences in the microbial composition in symptomatic uncomplicated diverticular disease patients compared to normal controls^10^. Our study was cross sectional. If we had found substantial differences in the bacterial composition of the diverticulosis subjects compared to controls, one might question whether the differences were a consequence of the diverticula and not a cause. In the absence of pronounced differences in composition, however, this is not a concern. The sensitivity of colonoscopy for diverticulosis is not known. Endoscopists in this study were aware of the study and were accompanied by a research assistant who prompted them to report diverticula in each colon segment. Consequently the sensitivity is likely to better than during a clinical exam, but some diverticula are likely to have been overlooked. However, in analyses where we included the number of diverticula, we still found no differences.

In summary, in a large study of individuals undergoing screening colonoscopy, we found little evidence of an association between adherent microbial communities and diverticulosis. Alterations in colon bacterial community composition are unlikely to be responsible for the development of colonic diverticulosis. Furthermore, the presence of diverticulosis does not appear to alter the microbial composition of the colon.

## Methods

### Participants

This cross-sectional study was designed to assess factors associated with colonic diverticulosis (NIH R01DK094738). Details of the study methods have been described previously^7,11^. Briefly, 226 case subjects with one or more diverticula and 309 controls without diverticula were drawn from outpatients undergoing first time screening colonoscopy at the Meadowmont Ambulatory Endoscopy Center, University of North Carolina Hospitals, Chapel Hill, North Carolina. The study included consented subjects 30 years and older who had satisfactory colonoscopy preparation and complete examination to the cecum. The study excluded those with a history of previous colon resection, or a prior diagnosis of polyposis, colitis, colon cancer, diverticulosis or diverticular disease.

Endoscopists carefully examined the colon for diverticula in all segments and the results were recorded on special data collection forms. The number of diverticula in each segment of the colon (cecum, ascending, transverse, descending, sigmoid) was recorded and the number summed to indicate the total number of diverticula observed. Biopsies were taken adjacent to sigmoid diverticula when present or from the mid sigmoid in subjects with no diverticula. The biopsies (approximately 3–4 mm in diameter)^21^ were obtained using standard (8 mm. wing) disposable, fenestrated colonoscopy forceps. Two biopsies obtained for microbiota profiling were rinsed in sterile PBS prior to freezing in liquid nitrogen to avoid contamination with fecal bacteria^22^. Laboratory personnel were blinded to clinical information and diverticulosis status of subjects. The study was approved by the University of North Carolina Office of Human Research Ethics. All participants gave informed consent. Enrollment of participants and laboratory experiments were performed in accordance with the relevant guidelines and institutional regulations.

### DNA Extraction, PCR and sequencing

We extracted bacterial genomic DNA from mucosal biopsy specimens as previously described^23,24^. Briefly, normal biopsies from each patient were placed in lysozyme for 30 minutes followed by bead beating and DNA extraction (Qiagen DNeasy Blood and Tissue, kit cat # 69504). The DNA fractions were eluted in 30 μl of elution buffer and stored in aliquots at −20 °C.

Illumina library creation was performed using two separate PCR reactions according to a previously published protocol^25^. The first-step PCR (PCR1) contained primers designed to amplify the V2 region of the 16S bacterial rRNA gene and Phusion High-Fidelity Master Mix (Life Technologies, Carlsbad, CA). PCR1 product was diluted 20-fold and used as a template for second-step PCR (PCR2). PCR2 primers contained an Illumina index barcode sequence, Illumina adapter sequence and a tag sequence. There were two sets of PCR2 primers, and each PCR2 reaction received one of each, resulting in a dual-indexed product. One reaction was performed for each sample using Phusion High-Fidelity Master Mix.

PCR product was visualized by E-Gel 96 to check samples for amplification. All samples with positive amplification were normalized to 25 ng/µl using the SequalPrep Normalization Kit (Life Technologies, Carlsbad, CA), and an equal volume of each sample library was pooled followed by cleaning using AxyPrep Mag Beads^25^. The pool was stored at −20 °C, then shipped to the University of Maryland Institute for Genome Sciences for sequencing using the Illumina MiSeq protocol^25^. Appropriate positive and negative controls were included in all sample preparation steps. A pooled sample of known bacteria served as positive control.

### Sequence processing and statistical analysis

Although producing adequate DNA can be challenging from biopsy samples, >90% of these samples had at least 1,000 reads assigned by different taxonomy algorithms (Table 4, Suppl. Figure 1) and these samples were used for downstream analysis at each taxonomic level. Forward reads were de-multiplexed and ran through version 2.10.1 of the RDP classification algorithm^26^. at a 50% confidence score (Table 1) or pick_closed_reference_otus.py script in QIIME 1.91. Read counts were log normalized as previously described^20^.

**Table 4:** Number of sequences identified by the RDP classification algorithm*.

Level	Total Sequences	Average	SD	Fraction Above1000
phylum	12,656,355	23,656.74	18,811.06	0.96
class	12,586,705	23,526.55	18,747.07	0.96
order	12,550,239	23,458.39	18,703.47	0.96
family	12,369,533	23,120.62	18,402.11	0.96
genus	11,362,538	21,238.39	17,050.57	0.96
OTU	8,240,210	15,402.26	12,869.20	0.93

*The number of sequences identified by the RDP classification algorithm at a threshold of 50% (for phylum through genus) or were assigned to an OTU in QIIME 1.91. Almost all 226 case and 309 control samples had at least 1,000 sequences per sample (last column) and these samples were used for analysis at each phylogenetic level.

The alpha-diversity and richness measurements were performed using the functions “diversity” and “rarefy” from the vegan package in R, with the subsample size of “rarefy” set to the minimum number of sequences detected in any sample. MDS ordination was performed with Bray-Curtis dissimilarity using the vegan package in R. Log-normalized abundance values for each taxon at the phyla, class, order, family and genus levels (RDP algorithm) or OTU were evaluated with a series of linear models and non-parametric tests. P-values were corrected for multiple hypothesis testing using B & H FDR correction^27^ with correction occurring separately for each test at each taxonomic level. To preserve power, statistical tests were only constructed for taxa that were present in at least 25% of all samples. All linear models and statistical tests were conducted in R. The R code used is available here: https://github.com/afodor/metagenomicsTools/blob/master/src/scripts/topeOneAtATime/metadataTests.txt

Each linear model took the form of:

1 $${\rm{Y}}={\rm{metadata}}+{\rm{error}}$$

Where “Y” is the alpha-diversity, richness, MDS axis or log normalized abundance and the metadata is the case/control status (for a two-factor one-way ANOVA), sex (for a two-factor one-way ANOVA), or race (white, black or other for a three-factor one-way ANOVA) or tics count (for a linear regression) or waist circumference (for a linear regression). As indicated in the text, non-parametric equivalents to linear models were used to generate p-values including the Wilcoxon test for two-factor metadata, Kruskal-Wallis test for multi-factor metadata, and the Kendall test for association of two quantitative variables.

In order to ensure that our results were not a consequence of our use of the RDP algorithm, we performed t-tests comparing case and control status for each taxa at the genus level with both the RDP algorithm and with the OTUs from the QIIME pipeline. The inference produced from these two classification schemes was highly concordant (Supplementary Fig. 1) demonstrating that our results are robust to our choice of classification scheme.

### Data Availability

The datasets generated from this study are available from the corresponding author on request. Raw sequences are available in the NCBI SRA data repository via submission SUB3467354 under Bioproject PRJNA429136.

## Electronic supplementary material

Supplementary Figure 1

Supplementary Tables 1-9
