# Supplementary material for: An Aberrant Microbiota is not Strongly Associated with Incidental Colonic Diverticulosis
Source: Sci Rep. 2018 Mar 21;8:4951. doi: 10.1038/s41598-018-23023-z (PMC5862835; doi:10.1038/s41598-018-23023-z)
Supplement: Supplementary file 1 — Supplementary Figure 1 [file 41598_2018_23023_MOESM1_ESM.docx]

**An Aberrant Microbiota is not Strongly Associated with Incidental Colonic Diverticulosis**

Roshonda B. Jones ^1^, Anthony A. Fodor ^1^, Anne F. Peery, ^2^ Matthew C. B. Tsilimigras ^1^, Kathryn Winglee ^1^, Amber McCoy, ^2^ Michael Sioda^1^ Robert S. Sandler^2^, Temitope O. Keku^2*^

^1^Department of Bioinformatics and Genomics, University of North Carolina, Charlotte, NC

^2^ Center for Gastrointestinal Biology and Disease, University of North Carolina, Chapel Hill, NC

Supplementary Fig. 1. Our results are not dependent on classification scheme.

Comparison of the results of a t-test evaluating a null hypothesis of equal distributions between case and control under RDP assignment to genus (x-axis) and Qiime closed reference assignment to genus (y-axis). Where control > case, the resulting p-value was multiplied by -1. Only taxa assigned under both classification schemes and present in at least 25% of all samples are shown. The close agreement on inference between the two pipelines suggests our results are not dependent on choice of classification scheme.
